# *N*′-[(*Z*)-4-(Dimethyl­amino)benzyl­idene]-4-nitro­benzohydrazide mono­hydrate

**DOI:** 10.1107/S1600536808028328

**Published:** 2008-09-13

**Authors:** Hoong-Kun Fun, Samuel Robinson Jebas, K. V. Sujith, P. S. Patil, B. Kalluraya

**Affiliations:** a X-ray Crystallography Unit, School of Physics, Universiti Sains Malaysia, 11800 USM, Penang, Malaysia; b Department of Studies in Chemistry, Mangalore University, Mangalagangotri, Mangalore 574199, India; c Department of Physics, K. L. E. Society’s K. L. E. Institute of Technology, Gokul Road, Hubli 590 030, India

## Abstract

In the asymmetric unit of the title compound, C_16_H_16_N_4_O_3_·H_2_O, there are two symmetry-independent hydrazide mol­ecules with almost identical geometries, and two independent water mol­ecules. The dihedral angles between the two benzene rings in the two hydrazide mol­ecules are 0.11 (5) and 0.77 (5)°. In one mol­ecule, an intra­molecular C—H⋯O hydrogen bond generates a ring of graph-set motif *S*(5). Inter­molecular N—H⋯O, O—H⋯O, O—H⋯N and C—H⋯O hydrogen bonds and π–π stacking inter­actions between the benzene rings [centroid–centroid distances in the range 3.5021 (6)–3.6403 (6) Å] are observed, together with O⋯O [2.7226 (11) Å], O⋯N [2.7072 (10) Å] and N⋯O [2.7072 (10)–2.8582 (12) Å] short contacts. The hydrazine mol­ecules are stacked along the *b* axis and adjacent mol­ecules are linked by water mol­ecules.

## Related literature

For related literature on hydrazones, see: Rollas & Küçükgüzel (2007 ▶); Singh *et al.* (1992 ▶); Ergenç & Günay (1998 ▶); Durgun *et al.* (1993 ▶). For a related structure, see: Fun *et al.* (2008 ▶). For bond-length data, see: Allen *et al.* (1987 ▶). For graph-set analysis of hydrogen bonding, see: Bernstein *et al.* (1995 ▶).
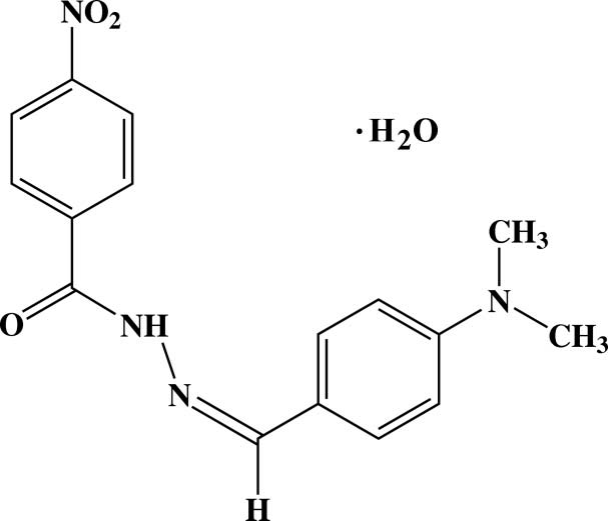


## Experimental

### 

#### Crystal data

C_16_H_16_N_4_O_3_·H_2_O
*M* *_r_* = 330.34
Triclinic, 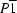

*a* = 6.5866 (2) Å
*b* = 7.1337 (2) Å
*c* = 34.4059 (12) Å
α = 92.113 (2)°
β = 90.918 (2)°
γ = 107.816 (1)°
*V* = 1537.42 (8) Å^3^
*Z* = 4
Mo *K*α radiation
μ = 0.10 mm^−1^
*T* = 100.0 (1) K
0.41 × 0.13 × 0.10 mm

#### Data collection

Bruker SMART APEXII CCD area-detector diffractometer
Absorption correction: multi-scan (*SADABS*; Bruker, 2005 ▶) *T*_min_ = 0.959, *T*_max_ = 0.990
52996 measured reflections
11039 independent reflections
8656 reflections with *I* > 2σ(*I*)
*R*_int_ = 0.028

#### Refinement

*R*[*F* ^2^ > 2σ(*F* ^2^)] = 0.048
*wR*(*F* ^2^) = 0.155
*S* = 1.07
11039 reflections
461 parameters
8 restraints
H atoms treated by a mixture of independent and constrained refinement
Δρ_max_ = 0.45 e Å^−3^
Δρ_min_ = −0.36 e Å^−3^

### 

Data collection: *APEX2* (Bruker, 2005 ▶); cell refinement: *APEX2*; data reduction: *SAINT* (Bruker, 2005 ▶); program(s) used to solve structure: *SHELXTL* (Sheldrick, 2008 ▶); program(s) used to refine structure: *SHELXTL*; molecular graphics: *SHELXTL*; software used to prepare material for publication: *SHELXTL* and *PLATON* (Spek, 2003 ▶).

## Supplementary Material

Crystal structure: contains datablocks global, I. DOI: 10.1107/S1600536808028328/is2331sup1.cif

Structure factors: contains datablocks I. DOI: 10.1107/S1600536808028328/is2331Isup2.hkl

Additional supplementary materials:  crystallographic information; 3D view; checkCIF report

## Figures and Tables

**Table 1:** Hydrogen-bond geometry (Å, °)

*D*—H⋯*A*	*D*—H	H⋯*A*	*D*⋯*A*	*D*—H⋯*A*
N1*A*—H1*NA*⋯O1*W*^i^	0.850 (9)	2.024 (10)	2.8582 (11)	166.8 (16)
O2*W*—H2*W*2⋯O1*W*^ii^	0.839 (8)	2.066 (9)	2.8892 (12)	166.9 (16)
O2*W*—H1*W*2⋯N2*B*^iii^	0.838 (8)	2.435 (11)	3.1989 (12)	151.8 (16)
O2*W*—H1*W*2⋯O1*B*^iii^	0.838 (8)	2.453 (13)	3.1535 (11)	141.6 (15)
O1*W*—H2*W*1⋯O1*A*^iv^	0.848 (9)	1.907 (10)	2.7227 (10)	160.9 (18)
O1*W*—H2*W*1⋯N2*A*^iv^	0.848 (9)	2.550 (16)	3.1072 (11)	124.2 (14)
N1*B*—H1*NB*⋯O2*W*^v^	0.859 (9)	2.073 (9)	2.9260 (12)	171.5 (16)
O1*W*—H1*W*1⋯O1*B*^iii^	0.842 (9)	1.997 (9)	2.8304 (12)	170.2 (17)
C1*A*—H1*AA*⋯O1*W*^i^	0.93	2.49	3.3025 (13)	146
C8*A*—H8*AA*⋯O1*W*^i^	0.93	2.51	3.2886 (13)	141
C1*B*—H1*BA*⋯O2*W*^v^	0.93	2.41	3.3276 (13)	169
C5*B*—H5*BA*⋯O1*B*	0.93	2.42	2.7555 (13)	101
C8*B*—H8*BA*⋯O2*W*^v^	0.93	2.48	3.2977 (14)	147
C15*A*—H15*C*⋯O2*B*^vi^	0.96	2.58	3.4738 (15)	156
C15*B*—H15*F*⋯O2*A*^vii^	0.96	2.58	3.4773 (15)	156

Symmetry codes: (i) 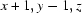; (ii) 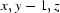; (iii) 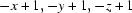; (iv) 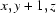; (v) 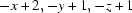; (vi) 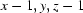; (vii) 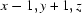.

**Fig. 1.**
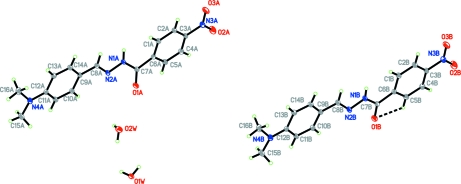
The molecular structure of the title compound, showing 50% probability displacement ellipsoids and the atom numbering scheme.

**Fig. 2.**
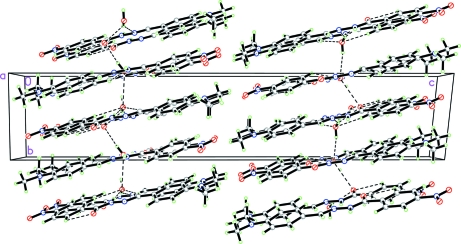
The crystal packing of the title compound, viewed down the a axis, showing infinite 1-D chains along the [010] direction.

## supplementary crystallographic information

### Comment

Hydrazones possessing an azometine —NHN═CH— proton constitute an important class of compounds for new drug development. Therefore, many researchers have synthesized these compounds as target structures and evaluated their biological activities. These observations have been the guides for the development of new hydrazones. Hydrazones containing an azometine —NHN═CH— proton are synthesized by heating the appropriate substituted hydrazines/hydrazides with aldehydes and ketones in solvents like ethanol, methanol, tetrahydrofuran, butanol, glacial acetic acid, ethanol-glacial acetic acid. Another synthetic route for the synthesis of hydrazones is the coupling of aryldiazonium salts with active hydrogen compounds (Rollas & Küçükgüzel, 2007). Hydrazide-hydrazones compounds are not only intermediates but they are also very effective organic compounds in their own right. When they are used as intermediates, coupling products can be synthesized by using the active hydrogen component of the —CONHN═CH— azometine group (Singh *et al.*, 1992). *N*-Alkyl hydrazides can be synthesized by reduction of hydrazones with NaBH_4_ (Ergenç & Günay, 1998), substituted 1,3,4-oxadiazolines can be synthesized when hydrazones are heated in the presence of acetic anhydride (Durgun *et al.*, 1993). Prompted by these review and in continuation of our work (Fun *et al.*, 2008), we herein report the crystal structure of the title compound, (I).

There are two independent molecules (A and B) in the asymmetric unit of (I), with similar geometries (Fig. 1.) The bond lengths and angles are found to have normal values (Allen *et al.*, 1987). The dihedral angle formed by the benzene (C1A–C6A) and (C9A–C14A) rings is 0.11 (5)° in molecule A and that between the benzene (C1B–C6B) and (C9B–C14B) rings is 0.77 (5)° in molecule B, indicating that they are coplanar. In molecule B, an intramolecular C—H···O hydrogen bond generates an S(5) ring motif (Bernstein *et al.*, 1995).

The crystal packing is consolidated by N—H···O, O—H···O, O—H···N and C—H···O inter and intramolecular hydrogen bonding (Table 1). Furthermore, the packing is strengthened by π–π stacking interactions involving the benzene (C1A–C6A) (*Cg*1) and the symmetry related (C9B–C14B) ring (*Cg*4) [*Cg*1···*Cg*4^i^ = 3.5021 Å; *Cg*1—*Cg*4^ii^ = 3.6403 (6) Å; symmetry codes: (i) 2-*x*, 1-*y*, 1-*z*; (ii) 2-*x*, -*y*, 1-*z*] and the benzene (C9A–C14A) ring (*Cg*2) and the symmetry related (C9B–C14B) ring (*Cg*3) [*Cg*2···*Cg*3^i^ = 3.6065 (6) Å; *Cg*2—*Cg*3^ii^ = 3.5274 (6) Å; symmetry codes: (i) 2-*x*, 1-*y*, 1-*z*; (ii) 2-*x*, -*y*, 1-*z*] together with O···O = 2.7226 (11) Å, O···N = 2.7072 (10) Å and N···O = 2.7072 (10)–2.8582 (12) Å short contacts. In the crystal packing, the molecules are stacked along the *b* axis and the adjacent molecules are linked by water molecules to form an infinite one dimensional chain along the [010] direction.

### Experimental

The title compound was obtained by refluxing 4-nitrrophenyl hydrazide (0.01 mol) and 4-(dimethylamino)benzaldehyde (0.01 mol) in ethanol (30 ml) with the addition of 3 drops of concentrated Sulfuric acid for 3 h. Excess ethanol was removed from the reaction mixture under reduced pressure. The solid product obtained was filtered, washed with water and dried. Crystals suitable for X-ray analysis were obtained from ethanol by slow evaporation.

### Refinement

The amino and water H atoms were located in a difference map and refined with restraints of N—H = 0.85 (1) and O—H = 0.84 (1) Å. The remaining H atoms were positioned geometrically [C—H = 0.93 (aromatic) or 0.96 Å (methyl)] and refined using a riding model, with *U*_iso_(H) = 1.2*U*_eq_(aromatic C) and 1.5*U*_eq_(methyl C). A rotating-group model was used for the methyl groups.

### Crystal data

**Table:**

C_16_H_16_N_4_O_3_·H_2_O	*Z* = 4
*M**_r_* = 330.34	*F*(000) = 696
Triclinic, *P*1	*D*_x_ = 1.427 Mg m^−^^3^
Hall symbol: -P 1	Mo *K*α radiation, λ = 0.71073 Å
*a* = 6.5866 (2) Å	Cell parameters from 9891 reflections
*b* = 7.1337 (2) Å	θ = 2.2–29.2°
*c* = 34.4059 (12) Å	µ = 0.11 mm^−^^1^
α = 92.113 (2)°	*T* = 100 K
β = 90.918 (2)°	Block, red
γ = 107.816 (1)°	0.41 × 0.13 × 0.10 mm
*V* = 1537.42 (8) Å^3^	

### Data collection

**Table:**

Bruker SMART APEXII CCD area-detector diffractometer	11039 independent reflections
Radiation source: fine-focus sealed tube	8656 reflections with *I* > 2σ(*I*)
graphite	*R*_int_ = 0.028
φ and ω scans	θ_max_ = 32.5°, θ_min_ = 0.6°
Absorption correction: multi-scan (SADABS; Bruker, 2005)	*h* = −9→9
*T*_min_ = 0.959, *T*_max_ = 0.990	*k* = −10→10
52996 measured reflections	*l* = −51→51

### Refinement

**Table:**

Refinement on *F*^2^	Primary atom site location: structure-invariant direct methods
Least-squares matrix: full	Secondary atom site location: difference Fourier map
*R*[*F*^2^ > 2σ(*F*^2^)] = 0.048	Hydrogen site location: inferred from neighbouring sites
*wR*(*F*^2^) = 0.155	H atoms treated by a mixture of independent and constrained refinement
*S* = 1.08	*w* = 1/[σ^2^(*F*_o_^2^) + (0.0736*P*)^2^ + 0.5006*P*] where *P* = (*F*_o_^2^ + 2*F*_c_^2^)/3
11039 reflections	(Δ/σ)_max_ = 0.001
461 parameters	Δρ_max_ = 0.45 e Å^−^^3^
8 restraints	Δρ_min_ = −0.36 e Å^−^^3^

### Special details

**Table:**

Experimental. The data was collected with the Oxford Cyrosystem Cobra low-temperature attachment.
Geometry. All e.s.d.'s (except the e.s.d. in the dihedral angle between two l.s. planes) are estimated using the full covariance matrix. The cell e.s.d.'s are taken into account individually in the estimation of e.s.d.'s in distances, angles and torsion angles; correlations between e.s.d.'s in cell parameters are only used when they are defined by crystal symmetry. An approximate (isotropic) treatment of cell e.s.d.'s is used for estimating e.s.d.'s involving l.s. planes.
Refinement. Refinement of *F*^2^ against ALL reflections. The weighted *R*-factor *wR* and goodness of fit *S* are based on *F*^2^, conventional *R*-factors *R* are based on *F*, with *F* set to zero for negative *F*^2^. The threshold expression of *F*^2^ > σ(*F*^2^) is used only for calculating *R*-factors(gt) *etc*. and is not relevant to the choice of reflections for refinement. *R*-factors based on *F*^2^ are statistically about twice as large as those based on *F*, and *R*- factors based on ALL data will be even larger.

### Fractional atomic coordinates and isotropic or equivalent isotropic displacement parameters (Å^2^)

**Table:**

	*x*	*y*	*z*	*U*_iso_*/*U*_eq_	
O1A	0.70471 (11)	−0.07582 (12)	0.30351 (2)	0.01918 (16)	
O2A	1.23137 (15)	−0.24154 (16)	0.46624 (3)	0.0307 (2)	
O3A	1.53442 (13)	−0.13214 (15)	0.43817 (3)	0.02754 (19)	
N1A	0.99847 (13)	−0.03980 (13)	0.26697 (2)	0.01355 (15)	
N2A	0.89267 (13)	−0.00979 (13)	0.23380 (2)	0.01426 (16)	
N3A	1.33845 (15)	−0.17619 (14)	0.43808 (3)	0.01864 (17)	
N4A	0.71656 (14)	0.20609 (14)	0.05749 (2)	0.01700 (17)	
C1A	1.24033 (15)	−0.04368 (15)	0.33741 (3)	0.01467 (17)	
H1AA	1.3181	0.0119	0.3161	0.018*	
C2A	1.34633 (15)	−0.06955 (15)	0.37105 (3)	0.01509 (18)	
H2AA	1.4945	−0.0333	0.3725	0.018*	
C3A	1.22662 (15)	−0.15040 (14)	0.40243 (3)	0.01401 (17)	
C4A	1.00511 (16)	−0.20815 (15)	0.40145 (3)	0.01574 (18)	
H4AA	0.9283	−0.2628	0.4229	0.019*	
C5A	0.90143 (15)	−0.18208 (14)	0.36758 (3)	0.01475 (17)	
H5AA	0.7532	−0.2192	0.3663	0.018*	
C6A	1.01742 (15)	−0.10065 (14)	0.33537 (3)	0.01233 (16)	
C7A	0.89305 (15)	−0.07167 (14)	0.30049 (3)	0.01324 (17)	
C8A	1.01208 (15)	0.04600 (14)	0.20449 (3)	0.01353 (17)	
H8AA	1.1575	0.0637	0.2074	0.016*	
C9A	0.92940 (15)	0.08245 (14)	0.16715 (3)	0.01250 (16)	
C10A	0.71074 (15)	0.03692 (14)	0.15792 (3)	0.01435 (17)	
H10A	0.6115	−0.0195	0.1764	0.017*	
C11A	0.64051 (15)	0.07458 (15)	0.12178 (3)	0.01456 (17)	
H11A	0.4948	0.0421	0.1164	0.017*	
C12A	0.78608 (15)	0.16157 (14)	0.09285 (3)	0.01284 (17)	
C13A	1.00565 (15)	0.20405 (15)	0.10204 (3)	0.01502 (17)	
H13A	1.1057	0.2589	0.0836	0.018*	
C14A	1.07347 (15)	0.16486 (15)	0.13832 (3)	0.01493 (17)	
H14A	1.2190	0.1941	0.1437	0.018*	
C15A	0.49080 (18)	0.14199 (19)	0.04745 (3)	0.0236 (2)	
H15A	0.4164	0.1962	0.0664	0.035*	
H15B	0.4376	0.0007	0.0471	0.035*	
H15C	0.4687	0.1865	0.0222	0.035*	
C16A	0.86549 (18)	0.26143 (18)	0.02608 (3)	0.0225 (2)	
H16A	0.9824	0.3745	0.0344	0.034*	
H16B	0.7939	0.2927	0.0039	0.034*	
H16C	0.9185	0.1537	0.0192	0.034*	
O1B	0.70699 (12)	0.38412 (13)	0.80186 (2)	0.02177 (17)	
O2B	1.25253 (15)	0.27622 (16)	0.96674 (3)	0.0316 (2)	
O3B	1.55367 (13)	0.37835 (15)	0.93778 (3)	0.02784 (19)	
N1B	1.01061 (13)	0.49010 (12)	0.76799 (2)	0.01420 (16)	
N2B	0.89598 (14)	0.51210 (12)	0.73516 (3)	0.01539 (16)	
N3B	1.35800 (15)	0.33755 (14)	0.93811 (3)	0.01923 (18)	
N4B	0.70414 (14)	0.70854 (14)	0.55794 (3)	0.01718 (17)	
C1B	1.25594 (15)	0.46004 (15)	0.83674 (3)	0.01543 (18)	
H1BA	1.3326	0.5104	0.8150	0.019*	
C2B	1.36291 (16)	0.43702 (15)	0.87055 (3)	0.01629 (18)	
H2BA	1.5110	0.4714	0.8716	0.020*	
C3B	1.24511 (16)	0.36207 (14)	0.90255 (3)	0.01464 (17)	
C4B	1.02391 (16)	0.30698 (15)	0.90212 (3)	0.01653 (18)	
H4BA	0.9482	0.2558	0.9239	0.020*	
C5B	0.91904 (16)	0.33052 (15)	0.86818 (3)	0.01572 (18)	
H5BA	0.7708	0.2946	0.8672	0.019*	
C6B	1.03316 (15)	0.40749 (14)	0.83543 (3)	0.01277 (16)	
C7B	0.90385 (15)	0.42761 (14)	0.80052 (3)	0.01407 (17)	
C8B	1.01092 (16)	0.56375 (14)	0.70506 (3)	0.01526 (18)	
H8BA	1.1568	0.5830	0.7074	0.018*	
C9B	0.92289 (15)	0.59328 (14)	0.66764 (3)	0.01363 (17)	
C10B	0.70382 (15)	0.54404 (14)	0.65874 (3)	0.01446 (17)	
H10B	0.6059	0.4873	0.6774	0.017*	
C11B	0.63122 (15)	0.57871 (15)	0.62253 (3)	0.01458 (17)	
H11B	0.4852	0.5445	0.6173	0.017*	
C12B	0.77534 (15)	0.66547 (14)	0.59329 (3)	0.01311 (17)	
C13B	0.99531 (15)	0.70948 (15)	0.60209 (3)	0.01521 (18)	
H13B	1.0941	0.7631	0.5834	0.018*	
C14B	1.06500 (15)	0.67355 (14)	0.63834 (3)	0.01509 (18)	
H14B	1.2108	0.7036	0.6435	0.018*	
C15B	0.47861 (18)	0.64283 (19)	0.54800 (3)	0.0242 (2)	
H15D	0.4035	0.6948	0.5672	0.036*	
H15E	0.4271	0.5014	0.5473	0.036*	
H15F	0.4555	0.6886	0.5229	0.036*	
C16B	0.85261 (18)	0.76642 (18)	0.52658 (3)	0.0222 (2)	
H16D	0.9674	0.8813	0.5349	0.033*	
H16E	0.7797	0.7956	0.5043	0.033*	
H16F	0.9088	0.6607	0.5198	0.033*	
O1W	0.41887 (12)	0.95772 (12)	0.24824 (2)	0.01787 (15)	
O2W	0.53340 (12)	0.38228 (13)	0.24783 (2)	0.02204 (17)	
H1NA	1.1237 (17)	−0.049 (3)	0.2650 (5)	0.037 (4)*	
H2W2	0.485 (2)	0.2591 (12)	0.2452 (5)	0.046 (5)*	
H1W2	0.433 (2)	0.429 (2)	0.2445 (5)	0.042 (5)*	
H2W1	0.529 (2)	0.962 (3)	0.2618 (4)	0.048 (5)*	
H1NB	1.1465 (14)	0.523 (2)	0.7656 (5)	0.037 (4)*	
H1W1	0.397 (3)	0.860 (2)	0.2324 (4)	0.045 (5)*	

### Atomic displacement parameters (Å^2^)

**Table:**

	*U*^11^	*U*^22^	*U*^33^	*U*^12^	*U*^13^	*U*^23^
O1A	0.0129 (3)	0.0311 (4)	0.0149 (3)	0.0085 (3)	−0.0005 (2)	0.0032 (3)
O2A	0.0315 (4)	0.0487 (6)	0.0149 (4)	0.0152 (4)	0.0023 (3)	0.0136 (4)
O3A	0.0207 (4)	0.0431 (5)	0.0213 (4)	0.0132 (4)	−0.0048 (3)	0.0048 (4)
N1A	0.0129 (3)	0.0204 (4)	0.0095 (3)	0.0083 (3)	−0.0012 (3)	0.0024 (3)
N2A	0.0162 (4)	0.0191 (4)	0.0093 (4)	0.0082 (3)	−0.0026 (3)	0.0015 (3)
N3A	0.0226 (4)	0.0238 (4)	0.0122 (4)	0.0109 (3)	−0.0022 (3)	0.0033 (3)
N4A	0.0198 (4)	0.0230 (4)	0.0093 (4)	0.0078 (3)	0.0004 (3)	0.0048 (3)
C1A	0.0142 (4)	0.0188 (4)	0.0115 (4)	0.0056 (3)	0.0002 (3)	0.0019 (3)
C2A	0.0142 (4)	0.0206 (4)	0.0118 (4)	0.0074 (3)	−0.0011 (3)	0.0009 (3)
C3A	0.0176 (4)	0.0157 (4)	0.0104 (4)	0.0077 (3)	−0.0020 (3)	0.0013 (3)
C4A	0.0173 (4)	0.0172 (4)	0.0135 (4)	0.0059 (3)	0.0017 (3)	0.0049 (3)
C5A	0.0140 (4)	0.0169 (4)	0.0134 (4)	0.0045 (3)	0.0003 (3)	0.0036 (3)
C6A	0.0138 (4)	0.0137 (4)	0.0107 (4)	0.0060 (3)	−0.0003 (3)	0.0016 (3)
C7A	0.0135 (4)	0.0147 (4)	0.0118 (4)	0.0048 (3)	−0.0007 (3)	0.0011 (3)
C8A	0.0143 (4)	0.0155 (4)	0.0115 (4)	0.0055 (3)	−0.0010 (3)	0.0015 (3)
C9A	0.0148 (4)	0.0135 (4)	0.0101 (4)	0.0056 (3)	−0.0003 (3)	0.0016 (3)
C10A	0.0146 (4)	0.0181 (4)	0.0106 (4)	0.0051 (3)	0.0007 (3)	0.0032 (3)
C11A	0.0131 (4)	0.0189 (4)	0.0119 (4)	0.0051 (3)	−0.0001 (3)	0.0031 (3)
C12A	0.0160 (4)	0.0140 (4)	0.0095 (4)	0.0058 (3)	0.0006 (3)	0.0017 (3)
C13A	0.0156 (4)	0.0178 (4)	0.0123 (4)	0.0056 (3)	0.0029 (3)	0.0037 (3)
C14A	0.0137 (4)	0.0178 (4)	0.0141 (4)	0.0058 (3)	0.0008 (3)	0.0028 (3)
C15A	0.0214 (5)	0.0349 (6)	0.0157 (5)	0.0103 (4)	−0.0034 (4)	0.0056 (4)
C16A	0.0262 (5)	0.0283 (5)	0.0126 (5)	0.0070 (4)	0.0032 (4)	0.0062 (4)
O1B	0.0149 (3)	0.0334 (4)	0.0173 (4)	0.0079 (3)	−0.0011 (3)	0.0005 (3)
O2B	0.0323 (5)	0.0511 (6)	0.0146 (4)	0.0157 (4)	0.0034 (3)	0.0141 (4)
O3B	0.0214 (4)	0.0426 (5)	0.0212 (4)	0.0120 (4)	−0.0036 (3)	0.0068 (4)
N1B	0.0150 (4)	0.0176 (4)	0.0107 (4)	0.0059 (3)	−0.0023 (3)	0.0025 (3)
N2B	0.0178 (4)	0.0160 (4)	0.0128 (4)	0.0060 (3)	−0.0045 (3)	0.0013 (3)
N3B	0.0235 (4)	0.0235 (4)	0.0130 (4)	0.0104 (3)	−0.0016 (3)	0.0036 (3)
N4B	0.0186 (4)	0.0228 (4)	0.0110 (4)	0.0071 (3)	0.0004 (3)	0.0043 (3)
C1B	0.0156 (4)	0.0191 (4)	0.0121 (4)	0.0056 (3)	0.0006 (3)	0.0035 (3)
C2B	0.0152 (4)	0.0206 (4)	0.0140 (4)	0.0068 (3)	−0.0005 (3)	0.0035 (3)
C3B	0.0193 (4)	0.0158 (4)	0.0104 (4)	0.0077 (3)	−0.0013 (3)	0.0022 (3)
C4B	0.0188 (4)	0.0180 (4)	0.0137 (4)	0.0064 (3)	0.0021 (3)	0.0037 (3)
C5B	0.0154 (4)	0.0178 (4)	0.0141 (4)	0.0050 (3)	0.0010 (3)	0.0028 (3)
C6B	0.0147 (4)	0.0128 (4)	0.0115 (4)	0.0055 (3)	−0.0011 (3)	0.0004 (3)
C7B	0.0158 (4)	0.0147 (4)	0.0121 (4)	0.0055 (3)	−0.0015 (3)	−0.0005 (3)
C8B	0.0173 (4)	0.0155 (4)	0.0135 (4)	0.0060 (3)	−0.0027 (3)	0.0013 (3)
C9B	0.0159 (4)	0.0131 (4)	0.0125 (4)	0.0054 (3)	−0.0014 (3)	0.0009 (3)
C10B	0.0165 (4)	0.0163 (4)	0.0106 (4)	0.0047 (3)	0.0004 (3)	0.0034 (3)
C11B	0.0143 (4)	0.0174 (4)	0.0122 (4)	0.0048 (3)	−0.0004 (3)	0.0025 (3)
C12B	0.0169 (4)	0.0138 (4)	0.0099 (4)	0.0064 (3)	0.0006 (3)	0.0017 (3)
C13B	0.0154 (4)	0.0171 (4)	0.0138 (4)	0.0056 (3)	0.0022 (3)	0.0031 (3)
C14B	0.0147 (4)	0.0162 (4)	0.0150 (4)	0.0055 (3)	−0.0003 (3)	0.0022 (3)
C15B	0.0203 (5)	0.0376 (6)	0.0161 (5)	0.0105 (4)	−0.0025 (4)	0.0065 (4)
C16B	0.0251 (5)	0.0280 (5)	0.0128 (5)	0.0062 (4)	0.0029 (4)	0.0057 (4)
O1W	0.0143 (3)	0.0264 (4)	0.0148 (3)	0.0091 (3)	−0.0012 (3)	0.0013 (3)
O2W	0.0165 (3)	0.0271 (4)	0.0234 (4)	0.0074 (3)	0.0002 (3)	0.0056 (3)

### Geometric parameters (Å, °)

**Table:**

O1A—C7A	1.2379 (11)	O3B—N3B	1.2317 (12)
O2A—N3A	1.2278 (12)	N1B—C7B	1.3475 (13)
O3A—N3A	1.2315 (12)	N1B—N2B	1.3888 (11)
N1A—C7A	1.3462 (12)	N1B—H1NB	0.859 (9)
N1A—N2A	1.3846 (11)	N2B—C8B	1.2899 (13)
N1A—H1NA	0.850 (9)	N3B—C3B	1.4650 (13)
N2A—C8A	1.2875 (12)	N4B—C12B	1.3753 (12)
N3A—C3A	1.4667 (12)	N4B—C15B	1.4454 (14)
N4A—C12A	1.3751 (12)	N4B—C16B	1.4527 (14)
N4A—C15A	1.4475 (14)	C1B—C2B	1.3911 (13)
N4A—C16A	1.4540 (13)	C1B—C6B	1.3985 (13)
C1A—C2A	1.3892 (13)	C1B—H1BA	0.9300
C1A—C6A	1.3983 (13)	C2B—C3B	1.3847 (14)
C1A—H1AA	0.9300	C2B—H2BA	0.9300
C2A—C3A	1.3838 (13)	C3B—C4B	1.3878 (14)
C2A—H2AA	0.9300	C4B—C5B	1.3888 (14)
C3A—C4A	1.3889 (13)	C4B—H4BA	0.9300
C4A—C5A	1.3884 (13)	C5B—C6B	1.3988 (13)
C4A—H4AA	0.9300	C5B—H5BA	0.9300
C5A—C6A	1.3972 (13)	C6B—C7B	1.4978 (13)
C5A—H5AA	0.9300	C8B—C9B	1.4524 (13)
C6A—C7A	1.4981 (13)	C8B—H8BA	0.9300
C8A—C9A	1.4505 (13)	C9B—C14B	1.4019 (13)
C8A—H8AA	0.9300	C9B—C10B	1.4025 (13)
C9A—C14A	1.4005 (13)	C10B—C11B	1.3847 (13)
C9A—C10A	1.4043 (13)	C10B—H10B	0.9300
C10A—C11A	1.3835 (13)	C11B—C12B	1.4189 (13)
C10A—H10A	0.9300	C11B—H11B	0.9300
C11A—C12A	1.4167 (13)	C12B—C13B	1.4107 (13)
C11A—H11A	0.9300	C13B—C14B	1.3817 (13)
C12A—C13A	1.4119 (13)	C13B—H13B	0.9300
C13A—C14A	1.3857 (13)	C14B—H14B	0.9300
C13A—H13A	0.9300	C15B—H15D	0.9600
C14A—H14A	0.9300	C15B—H15E	0.9600
C15A—H15A	0.9600	C15B—H15F	0.9600
C15A—H15B	0.9600	C16B—H16D	0.9600
C15A—H15C	0.9600	C16B—H16E	0.9600
C16A—H16A	0.9600	C16B—H16F	0.9600
C16A—H16B	0.9600	O1W—H2W1	0.848 (9)
C16A—H16C	0.9600	O1W—H1W1	0.842 (9)
O1B—C7B	1.2396 (12)	O2W—H2W2	0.839 (8)
O2B—N3B	1.2297 (12)	O2W—H1W2	0.838 (8)
			
C7A—N1A—N2A	118.95 (8)	C7B—N1B—H1NB	125.9 (11)
C7A—N1A—H1NA	122.1 (12)	N2B—N1B—H1NB	115.4 (11)
N2A—N1A—H1NA	118.7 (12)	C8B—N2B—N1B	113.95 (8)
C8A—N2A—N1A	114.85 (8)	O2B—N3B—O3B	123.26 (9)
O2A—N3A—O3A	123.44 (9)	O2B—N3B—C3B	118.35 (9)
O2A—N3A—C3A	118.16 (9)	O3B—N3B—C3B	118.39 (9)
O3A—N3A—C3A	118.40 (9)	C12B—N4B—C15B	119.95 (8)
C12A—N4A—C15A	119.77 (8)	C12B—N4B—C16B	119.70 (9)
C12A—N4A—C16A	119.76 (8)	C15B—N4B—C16B	117.92 (9)
C15A—N4A—C16A	117.83 (9)	C2B—C1B—C6B	120.04 (9)
C2A—C1A—C6A	120.39 (9)	C2B—C1B—H1BA	120.0
C2A—C1A—H1AA	119.8	C6B—C1B—H1BA	120.0
C6A—C1A—H1AA	119.8	C3B—C2B—C1B	118.87 (9)
C3A—C2A—C1A	118.55 (9)	C3B—C2B—H2BA	120.6
C3A—C2A—H2AA	120.7	C1B—C2B—H2BA	120.6
C1A—C2A—H2AA	120.7	C2B—C3B—C4B	122.54 (9)
C2A—C3A—C4A	122.60 (9)	C2B—C3B—N3B	118.80 (9)
C2A—C3A—N3A	118.59 (9)	C4B—C3B—N3B	118.65 (9)
C4A—C3A—N3A	118.82 (9)	C3B—C4B—C5B	118.02 (9)
C5A—C4A—C3A	118.18 (9)	C3B—C4B—H4BA	121.0
C5A—C4A—H4AA	120.9	C5B—C4B—H4BA	121.0
C3A—C4A—H4AA	120.9	C4B—C5B—C6B	120.93 (9)
C4A—C5A—C6A	120.71 (9)	C4B—C5B—H5BA	119.5
C4A—C5A—H5AA	119.6	C6B—C5B—H5BA	119.5
C6A—C5A—H5AA	119.6	C1B—C6B—C5B	119.59 (9)
C5A—C6A—C1A	119.57 (8)	C1B—C6B—C7B	124.01 (9)
C5A—C6A—C7A	117.17 (8)	C5B—C6B—C7B	116.40 (8)
C1A—C6A—C7A	123.22 (8)	O1B—C7B—N1B	122.42 (9)
O1A—C7A—N1A	123.57 (9)	O1B—C7B—C6B	120.50 (9)
O1A—C7A—C6A	120.16 (9)	N1B—C7B—C6B	117.07 (8)
N1A—C7A—C6A	116.26 (8)	N2B—C8B—C9B	122.99 (9)
N2A—C8A—C9A	122.77 (9)	N2B—C8B—H8BA	118.5
N2A—C8A—H8AA	118.6	C9B—C8B—H8BA	118.5
C9A—C8A—H8AA	118.6	C14B—C9B—C10B	117.73 (9)
C14A—C9A—C10A	117.64 (8)	C14B—C9B—C8B	118.20 (9)
C14A—C9A—C8A	118.88 (8)	C10B—C9B—C8B	124.06 (9)
C10A—C9A—C8A	123.48 (8)	C11B—C10B—C9B	120.92 (9)
C11A—C10A—C9A	121.09 (9)	C11B—C10B—H10B	119.5
C11A—C10A—H10A	119.5	C9B—C10B—H10B	119.5
C9A—C10A—H10A	119.5	C10B—C11B—C12B	121.27 (9)
C10A—C11A—C12A	121.35 (9)	C10B—C11B—H11B	119.4
C10A—C11A—H11A	119.3	C12B—C11B—H11B	119.4
C12A—C11A—H11A	119.3	N4B—C12B—C13B	121.07 (9)
N4A—C12A—C13A	121.29 (8)	N4B—C12B—C11B	121.46 (9)
N4A—C12A—C11A	121.36 (8)	C13B—C12B—C11B	117.46 (8)
C13A—C12A—C11A	117.34 (8)	C14B—C13B—C12B	120.49 (9)
C14A—C13A—C12A	120.62 (9)	C14B—C13B—H13B	119.8
C14A—C13A—H13A	119.7	C12B—C13B—H13B	119.8
C12A—C13A—H13A	119.7	C13B—C14B—C9B	122.08 (9)
C13A—C14A—C9A	121.94 (9)	C13B—C14B—H14B	119.0
C13A—C14A—H14A	119.0	C9B—C14B—H14B	119.0
C9A—C14A—H14A	119.0	N4B—C15B—H15D	109.5
N4A—C15A—H15A	109.5	N4B—C15B—H15E	109.5
N4A—C15A—H15B	109.5	H15D—C15B—H15E	109.5
H15A—C15A—H15B	109.5	N4B—C15B—H15F	109.5
N4A—C15A—H15C	109.5	H15D—C15B—H15F	109.5
H15A—C15A—H15C	109.5	H15E—C15B—H15F	109.5
H15B—C15A—H15C	109.5	N4B—C16B—H16D	109.5
N4A—C16A—H16A	109.5	N4B—C16B—H16E	109.5
N4A—C16A—H16B	109.5	H16D—C16B—H16E	109.5
H16A—C16A—H16B	109.5	N4B—C16B—H16F	109.5
N4A—C16A—H16C	109.5	H16D—C16B—H16F	109.5
H16A—C16A—H16C	109.5	H16E—C16B—H16F	109.5
H16B—C16A—H16C	109.5	H2W1—O1W—H1W1	106.7 (12)
C7B—N1B—N2B	118.62 (8)	H2W2—O2W—H1W2	108.6 (12)
			
C7A—N1A—N2A—C8A	170.93 (9)	C7B—N1B—N2B—C8B	−176.98 (9)
C6A—C1A—C2A—C3A	−0.75 (15)	C6B—C1B—C2B—C3B	−0.12 (15)
C1A—C2A—C3A—C4A	0.60 (15)	C1B—C2B—C3B—C4B	0.67 (16)
C1A—C2A—C3A—N3A	−179.48 (9)	C1B—C2B—C3B—N3B	179.87 (9)
O2A—N3A—C3A—C2A	177.47 (10)	O2B—N3B—C3B—C2B	177.95 (10)
O3A—N3A—C3A—C2A	−2.85 (15)	O3B—N3B—C3B—C2B	−2.44 (15)
O2A—N3A—C3A—C4A	−2.60 (15)	O2B—N3B—C3B—C4B	−2.82 (15)
O3A—N3A—C3A—C4A	177.07 (10)	O3B—N3B—C3B—C4B	176.80 (10)
C2A—C3A—C4A—C5A	−0.35 (15)	C2B—C3B—C4B—C5B	−0.61 (16)
N3A—C3A—C4A—C5A	179.73 (9)	N3B—C3B—C4B—C5B	−179.81 (9)
C3A—C4A—C5A—C6A	0.25 (15)	C3B—C4B—C5B—C6B	0.00 (15)
C4A—C5A—C6A—C1A	−0.42 (15)	C2B—C1B—C6B—C5B	−0.46 (15)
C4A—C5A—C6A—C7A	−178.04 (9)	C2B—C1B—C6B—C7B	179.70 (9)
C2A—C1A—C6A—C5A	0.68 (15)	C4B—C5B—C6B—C1B	0.53 (15)
C2A—C1A—C6A—C7A	178.15 (9)	C4B—C5B—C6B—C7B	−179.62 (9)
N2A—N1A—C7A—O1A	−0.01 (15)	N2B—N1B—C7B—O1B	1.99 (15)
N2A—N1A—C7A—C6A	−179.29 (8)	N2B—N1B—C7B—C6B	−179.45 (8)
C5A—C6A—C7A—O1A	16.42 (14)	C1B—C6B—C7B—O1B	−177.60 (10)
C1A—C6A—C7A—O1A	−161.10 (10)	C5B—C6B—C7B—O1B	2.56 (14)
C5A—C6A—C7A—N1A	−164.26 (9)	C1B—C6B—C7B—N1B	3.81 (14)
C1A—C6A—C7A—N1A	18.21 (14)	C5B—C6B—C7B—N1B	−176.03 (9)
N1A—N2A—C8A—C9A	179.26 (8)	N1B—N2B—C8B—C9B	179.47 (9)
N2A—C8A—C9A—C14A	172.55 (9)	N2B—C8B—C9B—C14B	171.95 (9)
N2A—C8A—C9A—C10A	−8.37 (15)	N2B—C8B—C9B—C10B	−8.92 (16)
C14A—C9A—C10A—C11A	−0.81 (15)	C14B—C9B—C10B—C11B	−1.69 (14)
C8A—C9A—C10A—C11A	−179.90 (9)	C8B—C9B—C10B—C11B	179.17 (9)
C9A—C10A—C11A—C12A	−0.44 (15)	C9B—C10B—C11B—C12B	−0.18 (15)
C15A—N4A—C12A—C13A	173.52 (10)	C15B—N4B—C12B—C13B	173.26 (10)
C16A—N4A—C12A—C13A	12.30 (15)	C16B—N4B—C12B—C13B	11.36 (15)
C15A—N4A—C12A—C11A	−7.65 (15)	C15B—N4B—C12B—C11B	−7.93 (15)
C16A—N4A—C12A—C11A	−168.87 (9)	C16B—N4B—C12B—C11B	−169.83 (9)
C10A—C11A—C12A—N4A	−177.42 (9)	C10B—C11B—C12B—N4B	−176.95 (9)
C10A—C11A—C12A—C13A	1.46 (15)	C10B—C11B—C12B—C13B	1.91 (15)
N4A—C12A—C13A—C14A	177.65 (9)	N4B—C12B—C13B—C14B	177.09 (9)
C11A—C12A—C13A—C14A	−1.23 (14)	C11B—C12B—C13B—C14B	−1.76 (14)
C12A—C13A—C14A—C9A	0.00 (15)	C12B—C13B—C14B—C9B	−0.10 (15)
C10A—C9A—C14A—C13A	1.04 (15)	C10B—C9B—C14B—C13B	1.84 (15)
C8A—C9A—C14A—C13A	−179.83 (9)	C8B—C9B—C14B—C13B	−178.97 (9)

### Hydrogen-bond geometry (Å, °)

**Table:**

*D*—H···*A*	*D*—H	H···*A*	*D*···*A*	*D*—H···*A*
N1A—H1NA···O1W^i^	0.85 (1)	2.02 (1)	2.8582 (11)	167.(2)
O2W—H2W2···O1W^ii^	0.84 (1)	2.07 (1)	2.8892 (12)	167.(2)
O2W—H1W2···N2B^iii^	0.84 (1)	2.44 (1)	3.1989 (12)	152.(2)
O2W—H1W2···O1B^iii^	0.84 (1)	2.45 (1)	3.1535 (11)	142.(2)
O1W—H2W1···O1A^iv^	0.85 (1)	1.91 (1)	2.7227 (10)	161.(2)
O1W—H2W1···N2A^iv^	0.85 (1)	2.55 (2)	3.1072 (11)	124.(1)
N1B—H1NB···O2W^v^	0.86 (1)	2.07 (1)	2.9260 (12)	172.(2)
O1W—H1W1···O1B^iii^	0.84 (1)	2.00 (1)	2.8304 (12)	170.(2)
C1A—H1AA···O1W^i^	0.93	2.49	3.3025 (13)	146
C8A—H8AA···O1W^i^	0.93	2.51	3.2886 (13)	141
C1B—H1BA···O2W^v^	0.93	2.41	3.3276 (13)	169
C5B—H5BA···O1B	0.93	2.42	2.7555 (13)	101
C8B—H8BA···O2W^v^	0.93	2.48	3.2977 (14)	147
C15A—H15C···O2B^vi^	0.96	2.58	3.4738 (15)	156
C15B—H15F···O2A^vii^	0.96	2.58	3.4773 (15)	156

Symmetry codes: (i) *x*+1, *y*−1, *z*; (ii) *x*, *y*−1, *z*; (iii) −*x*+1, −*y*+1, −*z*+1; (iv) *x*, *y*+1, *z*; (v) −*x*+2, −*y*+1, −*z*+1; (vi) *x*−1, *y*, *z*−1; (vii) *x*−1, *y*+1, *z*.
